# Serum MicroRNA-196, -200 and -423 Improve Diagnostics and Differentiate Pancreatic Ductal Adenocarcinoma From Chronic Pancreatitis

**DOI:** 10.33549/physiolres.935586

**Published:** 2025-10-01

**Authors:** Pavel ŠKRHA, Aleš HOŘÍNEK, Jan HAJER, Jana POTOČKOVÁ, Přemysl FRIČ, Jan BUREŠ, Michal ANDĚL, Jan ŠKRHA

**Affiliations:** 1 Second Department of Internal Medicine, Third Faculty of Medicine, Charles University, Faculty Hospital Královské Vinohrady, Prague, Czech Republic; 2 Third Department of Internal Medicine, First Faculty of Medicine, Charles University, General University Hospital, Prague, Czech Republic; 3 Department of Internal Medicine, First Faculty of Medicine, Charles University, Military University Hospital, Prague, Czech Republic

**Keywords:** microRNA, Pancreatic ductal adenocarcinoma, Chronic pancreatitis, Biomarker, CA19-9

## Abstract

Sustained poor survival rate in pancreatic ductal adenocarcinoma (PDAC) calls for an earlier diagnosis to assure curative treatment. New powerful biomarkers are necessary because the currently used CA19-9 is not sensitive enough to distinguish PDAC, especially from chronic pancreatitis (CP). Expressions of miRNA-21, -30 -192, -196, -200, and -423 were measured in 77 patients with PDAC, 26 patients with CP and 64 non-cancer/non-CP subjects (39 patients with type 2 diabetes mellitus and 25 control healthy persons). Eleven patients with PDAC had CP at the background. The expressions of all microRNAs were significantly 1.4–3.7 times higher in the PDAC group compared to non-cancer/non-CP subjects and 2.2–6.1 times higher compared to CP patients. No difference in miRNA expressions was found between diabetic and non-diabetic patients. CA19-9 did not distinguish CP from PDAC patients with the history of CP, whereas all six miRNAs were able to do it. Adding miR-196, -200 and -423 to current marker CA19-9 improved sensitivity by 7 % (to 93 %) and specificity by 8 % (to 89 %). MicroRNA-423 could significantly distinguish PDAC from CP with both sensitivity and specificity 96 %. Panel of six miRNAs could be used as reliable marker in differentiating PDAC from chronic pancreatitis with the most impressive difference in miR-196 and miR-423.

## Introduction

Pancreatic ductal adenocarcinoma (PDAC) remains one of the most lethal cancer in humans. Although the estimated incidence of PDAC in the USA for 2018 is not the highest among other cancer types it ranks the 3^rd^ place of all estimated cancer related deaths [1]. There are several risk factors in developing PDAC, such as alcoholism, smoking, diabetes mellitus, obesity, or liver cirrhosis, but one of the most important is chronic pancreatitis (CP), that increases the risk 1.8–4 times [2]. This risk can be caused by frequent association of CP with other risk factors of PDAC.

Diagnosis of PDAC is rather difficult in the initial stages when no signs or symptoms are present. PDAC is therefore frequently confirmed in later stages when dissemination of the cancer already exists. The widely used current marker CA19-9 is not sensitive enough to distinguish PDAC from CP. New biomarkers should be introduced to confirm diagnosis of PDAC with higher specificity and sensitivity still before examination of patients by high resolution imaging methods.

Number of different biomarkers, i.e. DNA mutations and methylations, RNA, proteins, circulating tumor cells and multiomics analyses have been tested but no final recommendation was suggested until now [3]. Promising results have been done by lipidomic analysis distinguishing patients with PDAC from healthy persons [4]. However, further research in this field is still necessary to confirm first analysis. MicroRNAs (miRNAs) showed a new potential in diagnosis and treatment of different tumors including PDAC [5,6]. Different gene expression of miRNAs in pancreatic tissue was found in patients with PDAC compared to healthy persons [7]. Dysregulated miRNAs in pancreatic cancer may explain the changes in key signaling pathways participating in cell cycle control, proliferation, differentiation, apoptosis and metastasis [8]. Changes in plasma miRNAs corresponding with tissue expressions stimulate their investigation like new biomarkers [9,10]. Large number of miRNAs has been tested in pancreatic ductal adenocarcinoma and several of them have been suggested as reliable diagnostic and prognostic biomarkers [9,11]. In addition, miRNAs play significant role in progression and metastases of pancreatic cancer [12]. They may be used as markers of successful treatment as well [13].

We found two microRNAs, miR-196 and miR-200, that were able to distinguish PDAC from the non-cancer group [14]. PDAC is frequently associated with both long-term and recent-onset diabetes mellitus but we did not find any statistically significant difference in miRNAs among the subgroups according to the presence or absence of diabetes [14].

Different expression of miRNAs was found in pancreatic tissue of patients with PDAC and CP and some studies demonstrated miRNA changes in serum samples [15,16]. Some novel miRNAs have been found in PDAC differently from CP [15] and they may support differentiation between both diseases.

In our study we compare six miRNAs and CA19-9 in patients with CP without PDAC and in patients with histologically approved PDAC and compare them with non-cancer control subjects.

## Patients and Methods

### Study population

Seventy-seven patients with PDAC, 26 with chronic pancreatitis and 25 healthy controls were examined in this study. Another group of 39 patients with Type 2 diabetes mellitus without any symptoms or signs of malignant disease or chronic pancreatitis was used as a second control according to our previous results [14].

Eleven patients with PDAC (14 %) had CP at the background. Sixty patients with PDAC (78 %) had impaired glucose homeostasis classified as prediabetes or diabetes. Seventeen patients of them had long-term type 2 diabetes mellitus and the remaining 43 developed prediabetes or diabetes up to 24 months prior to diagnosis of PDAC. This new-onset diabetes (T3cDM) was the first manifestation of pancreatic cancer. Diagnosis of diabetes mellitus was confirmed by standard criteria according to recommendations of American Diabetes Association [17].

Patients with CP were enrolled from the outpatient department of gastroenterology. Diagnosis of CP was based on the case history of patients and results of imaging methods (computed tomography, endoscopic ultrasound or endoscopic retrograde cholangio-pancreatography). The etiology of CP was ethanol-induced or recurrent acute pancreatitis.

The diagnosis of PDAC was confirmed by a biopsy or after surgical resection. In 70 of 77 patients with PDAC the advanced stages (T3 or T4) of cancer were present whereas T1 or T2 were in the remaining seven. Characteristics of the groups are shown in Table 1. Patients with T2DM had higher BMI compared to patients with CP and PDAC associated with diabetes. 52 patients with PDAC (68 %) had a significant body weight loss (more than 5 % in 3 months or more than 10 % in 6 months) before the diagnosis. All subjects agreed and subscribed an informed consent. The study protocol was approved by the local Ethics Committee, according to the principles outlined in the Declaration of Helsinki.

### Laboratory methods

All laboratory examinations were done in the fasting state. Blood samples were drawn between 7.00 and 8.00 a.m., serum was separated by centrifugation after 30 min of blood coagulation and stored at −70 °C. Fasting plasma glucose (FPG), glycated hemoglobin (HbA1c) and CA19-9 were determined by routine tests in the central laboratory (Table 1). Chemiluminiscence method was used for CA19-9 estimation performed on Architect analyzer (Abbott).

Frozen serum (−70 °C) was used for the analyses of miRNAs. Reverse transcription and real-time PCR (using 7900HT Fast Real-Time PCR System) with individual assays using single TaqMan® MicroRNA Assays (Applied Biosystems, Thermo Fisher Scientific, Waltham, MA, USA) were performed after miRNAs extraction using the miRCURY RNA Isolation Kit (Exiqon, Denmark). The raw results from the real-time PCR are numbers of cycles until reaching the automatic threshold set by the original software of the cycler. These results (raw expression data) were processed in Expression Suite Software v1.0.3 (Applied Biosystems, Thermo Fisher Scientific, Waltham, MA, USA) and 8 miRNAs (miR-21, miR-30, miR-191, miR-192, miR-196, miR-200, miR-423 and miR-454) were analyzed. Two miRNAs (miR-191, miR-454), chosen by the geNorm analysis within the qBase+© program v2.4 (Biogazelle, Belgium), were used for the normalization of miRNA expression rates for all the parts of the study. The mean expression of these two miRNA normalizers was subtracted from the respective miRNA expression, difference of which was its relative fold change. Results of miRNAs are thus expressed in relative units as a fold change from the normalizers. miRNAs in all studied groups were expressed as a fold change related to the control group which was calculated as equal to one.

### Statistical evaluation and calculation

MiRNA results were analyzed by Kruskal-Wallis analysis of variance (ANOVA) with multiple comparison. Correlations between variables were assessed by Pearson’s coefficient with 95 % confidence interval (CI). For statistical analyses, “Statistica 12” software by StatSoft Inc. was used. The results were considered as statistically significant at p<0.05. The results were expressed as geometric means with SD ranges. Estimation of sensitivity and specificity was calculated by using ROC analysis [18].

## Results

### miRNAs in the separate groups

**Fig. 1:**
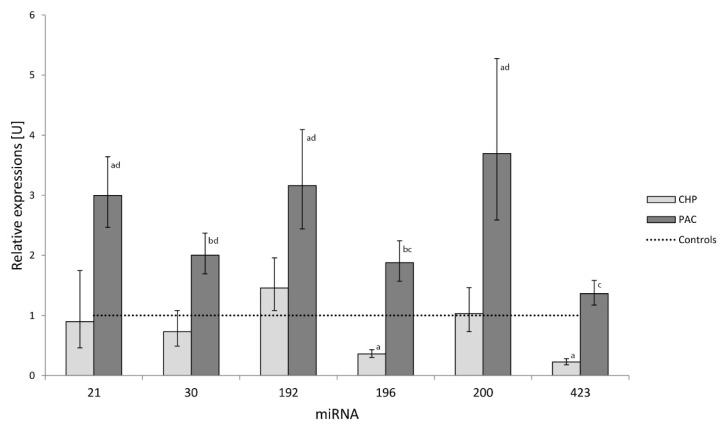
Relative miRNA expressions of PDAC (n=77) and chronic pancreatitis (n=26) patients compared to healthy persons expressed as equal to 1 (dotted line). Statistical significance: PDAC/CP vs. Controls (^a^ p<0.001, ^b^ p<0.01), PDAC vs. CP (^c^ p<0.001, ^d^ p<0.01).

Significant difference was found in the expressions of all tested miRNAs between PDAC and CP groups (Fig. 1). The results of individual miRNAs in patients with PDAC, CP and patients with T2DM were compared with the controls (Table 2). No significant difference was found in miRNA results between the control group of healthy persons and patients with T2DM. Significant differences among all three groups (PDAC, CP and patients with T2DM/healthy controls) were found only in miR-196, with the highest expressions in the PDAC patients. In PDAC the miR-196 was 5.2 and 1.9 times higher compared to that in CP and controls, respectively. Expressions of miR-423 were significantly lower (6.1 and 4.4 times) in CP compared to PDAC and the control group, respectively. However, expressions of miR-423 was not different in PDAC from the control group. Differences in the remaining evaluated miRNAs were significant between PDAC and CP patients and between PDAC and control group.

### miRNAs and diabetes mellitus in groups of patients with PDAC and CP

The influence of diabetes on biochemical markers and on miRNA expressions is shown in the Table 2. No significant difference in selected miRNAs was observed in the subgroups of patients with and without diabetes. However, difference in miRNAs was left between PDAC without diabetes and CP either with or without diabetes apart from miR-196 and miR-423. They were the only markers with persisting significant differences between CP and PDAC subgroups according to the presence or absence of diabetes.

### miRNAs in patients with PDAC and history of CP

**Fig. 2:**
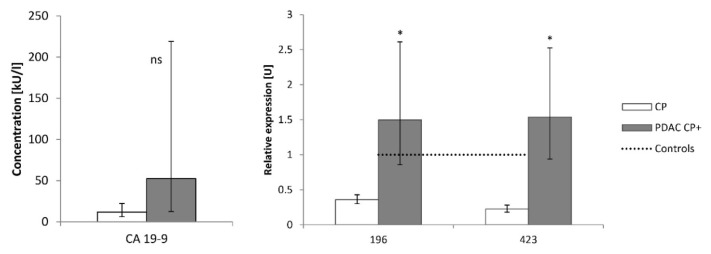
Comparison of CA19-9 concentrations and miR-196 and miR-423 expressions in patients with chronic pancreatitis and PDAC with the previous history of chronic pancreatitis. CP – chronic pancreatitis (n=26), PDAC CP^+^ – patients with pancreatic ductal adenocarcinoma with the history of chronic pancreatitis (n=11). Concentrations of CA19-9 are expressed as geometric means with 95 % CI, relative miRNA expressions are expressed as fold-change compared to the control group expressed as equal to 1 (dotted line). Statistical difference: PDAC vs. CP * p<0.001.

No difference in the miRNAs was observed between PDAC patients with previous history of CP and those without pancreatitis (data not shown). CA19-9 could not differentiate patients with CP from patients with PDAC with the previous history of pancreatitis whereas the expressions of both miR-196 and miR-423 showed the most significant difference of six miRNAs studied (Fig. 2). miR-423 was significantly reduced in non-cancer patients with CP compared to control and PDAC groups. No difference was observed in miR-423 expressions between control persons and patients with PDAC and previous history of CP.

### miRNAs in stages T1 to T4 of PDAC

**Fig. 3:**
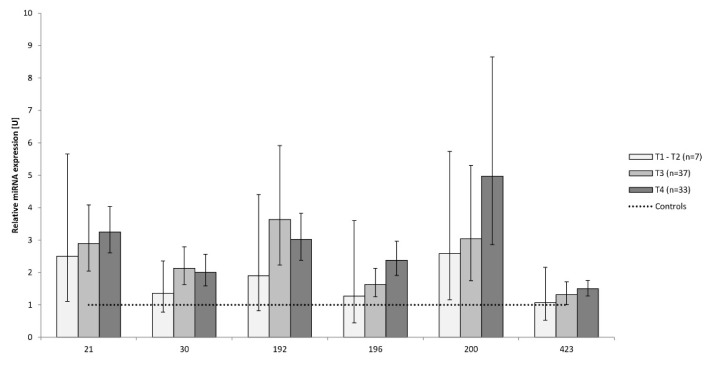
Relative expressions of miRNAs according to the stage of the pancreatic ductal adenocarcinoma compared to the control group expressed as equal to 1 (dotted line). Number of patients: T1–T2=7, T3=37, T4=33. T1 – T2 the tumor reaches the edges of the pancreas. T3 – tumor exceeds the edges of the pancreas and can be very close to the truncus coeliacus or a. mesenterica superior, but does not touch it. T4 – the tumor grows around truncus coeliacus or a. mesenterica superior. No significant differences were found among the stages of PDAC.

The results of miRNA expressions were compared in stages of the cancer (Fig. 3). There were only 7 patients with the stage of the cancer lower than T3 (1 patient with T1, 6 patients with T2), 37 patients with T3 and 33 patients with the stage T4. We could not find any difference in miRNAs between stages of the cancer, but their mean expressions have been increased from T1/T2 to T4.

### Sensitivity and specificity of CA19-9 and in combination with miRNAs

While CA19-9 sensitivity and specificity to differentiate patients with the cancer from non-cancer patients (controls, T2DM and CP) was 86 % and 81 %, respectively, the combined test of CA19-9, miR-196, -200 and -423 reached sensitivity and specificity of 93 % and 89 %. Sensitivity of CA19-9 remained the same and specificity dropped to 69 % when distinguishing PDAC only from CP patients who often have false positive results. On the other hand, sensitivity and specificity of miR-423 in the same setting was 96 % and 96 %, respectively.

## Discussion

High mortality rate of PDAC is caused by the late diagnosis, when the curative resection of the cancer, as the only successful treatment option, is not possible. Lack of suitable biomarkers and lack of symptoms in PDAC patients cause late diagnosis. Structural program for early detection of PDAC was suggested [19]. The new-onset diabetes (T3cDM) as the first manifestation of pancreatic cancer and the loss of weight can be used by primary care physicians. More patients with new-onset T2DM than T3cDM are in the general population and differentiation of both types is necessary [20]. The loss of weight is therefore an important symptom for screening.

We confirmed in our study that only few patients were diagnosed in the initial PDAC stages and demonstrated the important impact of new-onset diabetes for the early diagnosis (5 out of 7 patients with initial PDAC stages had T3cDM). Only 7.7 % of our PDAC patients with new-onset diabetes and weight loss had the initial stage of the cancer. Surgery can be offered to 15–20 % of patients with PDAC and relapses are frequent. Five-year survival rate after the resection is around 20–25 %, depending on the stage of the cancer and negativity of histological findings in the resection margins (R0 resection) [21,22]. The early detection of PDAC is therefore crucial.

### miRNAs and CA19-9 in diagnosis of PDAC

Our study demonstrated that panel using six miRNAs-21, -30, -192 -196, -200, and -423 with CA19-9 significantly increased both sensitivity (93 %) and specificity (89 %) of PDAC diagnosis. Sensitivity of 68–78 % and specificity of 70–83 % for the current marker CA 19-9 alone is quite low in pancreatic cancer [23,24]. Excellent results were recently confirmed in a large study analyzing data of one hundred miRNA together with CA19-9 [25]. This combination discriminated pancreatic cancer from healthy control with high accuracy 0.99, sensitivity 90 % and specificity 98 %. In addition, combination of miRNAs with CA19-9 could discriminate the early stages (stage 0–1) of cancer from healthy controls as well.

One retrospective study reported that microRNAs appear late in PDAC. No difference was found in miRNAs between the cancer and control subjects examined <5 years before the cancer diagnosis, while there was already a borderline elevation of CA19-9 (p=0.044) [26]. Although we did not make a retrospective analysis, correct marking of all initial stages of the cancer, using combined test with our selected miRNAs, shows, that miRNAs could play a role in earlier diagnosis of PDAC. This is supported by findings that miR-196 is significantly elevated even in precursors of PDAC, such as in pancreatic intraepithelial neoplasms (PanIN) 2–3 [27]. The miRNA elevations in PanINs were normalized after the surgery [28]. Similar finding was reported in other PDAC precursor, intraductal papillary mucinous neoplasms (IPMN) [29].

### miRNAs in differentiation of PDAC from chronic pancreatitis

Secondly, we could differentiate PDAC from CP using set of six miRNAs -21, -30, -192, -196, -200, and -423. The expressions of miR-21, miR-192 and miR-200 significantly distinguished PDAC from healthy controls. Differences between PDAC and CP groups were significant in all six miRNAs. However, only expression of miRNA-196 and miRNA-423 were significantly lower in CP as compared to the control group. This downregulation was found for the first time and no similar data have been published with these two miRNAs yet. We speculate that decreased expression of miR-196 and miR-423 could be used as a marker of CP however further studies will be necessary to confirm this idea. Dysregulation of miR-423 has been studied in different cancers [30]. It was classified as a critical factor in tumorigenesis and suggested as important biomarker in the early diagnosis and prognosis of human cancers. Downregulation of miR-423 was found in the latest article on PDAC patients in which the biosenzor was used for parallel detection of six miRNAs [31]. Other two miR-15 and miR-145 were found downregulated in PDAC but the article was oriented more technically on new methodology than to analyze clinical relationship.

MicroRNAs in patients with CP and PDAC were compared using several panels which discriminated both groups comparably to CA19-9 [32]. Two miRNAs, miR-486-5p and miR-938 were able to differentiate PDAC from healthy controls and CP with good areas under the curve (0.861 and 0.706; and 0.693 and 0.754 respectively) and both had diagnostic accuracy comparable with CA19-9 but their combination was not investigated [33]. Combination of two miRNAs, miR-16 and miR-196a possessed an independent role in discriminating PDAC from normal and CP subjects. The combination of miR-16, miR-196a and CA19-9 was more effective for discriminating PDAC from non-PDAC (normal+chronic pancreatitis) patients (sensitivity, 92.0 %; specificity, 95.6 %), and for discriminating PDAC from CP (sensitivity, 88.4 %; specificity, 96.3 %) [34]. Combination of miRNA with CA19-9 is therefore highly recommended for discrimination of PDAC from CP as well as for early diagnosis of PDAC [16].

Panel of eight serum miRNAs demonstrated that four miRNAs were upregulated and four downregulated in patients with PDAC [35]. Five miRNAs could differentiate PDAC from CP and healthy controls. Expressions of three miRNAs gradually increased from healthy controls to CP and to PDAC whereas the expression of one miRNA steadily decreased. The authors found differences in the expression of miRNAs between PDAC tissue and serum levels. [35]. This observation mentioned also by other investigators brings new insights on miRNA selection as biomarker for specific disease [36]. Concordance involving either upregulation or downregulation of the respective miRNAs in serum and tissue will approve their use in diagnosis and treatment.

### miRNAs and regulation of pathways in tissues

The differentiation of CP from PDAC is of great importance because it may influence the follow up of the patient. The elucidation of pathways regulated by miRNAs in both diseases is highlighted in the recent publication [37]. The PI3K/AKT/mTOR pathway was the most promising for CP versus healthy individuals whereas the TNF-alpha signaling is critical in PDAC patients. miR-130 and miR-148 have been identified as specific for PDAC, miR-192 and miR-150 for CP and miR-222 for differentiating PDAC from CP [37].

New data were obtained with exosomes (or extracellular vesicles) which transport specific molecules from maternal cells including miRNAs. Their perspectives are both in diagnosis and the treatment of PDAC patients where they bring new potential for drug delivery [38,39]. Exosomes transporting miRNAs and other biomolecules influence cellular processes like proliferation, angiogenesis, invasion and metastasis but also the resistance to some effective drugs like cisplatin, doxorubicine, 5-fluorouracil [8,40]. In addition, they play important role in development of T3c diabetes mellitus as an early sign of PDAC development [41]. More analyses with exosomes will be necessary to evaluate their use in clinical practice.

According to miRWalk database, which provides miRNA-target interactions (including validated interactions), one of the miR-196 targets is *PALLD* gene that encodes protein palladin. Palladin plays an important role in cellular morphogenesis and cell motility by regulation of actin cytoskeleton [42]. Overexpression of *palladin* RNA was observed both in familial and sporadic cancer. Transfection of mutated gene into the cell culture caused cytoskeletal changes with an increased ability to migration [43]. Salaria *et al.* found that the palladin protein overproduction is present mostly in the non-neoplastic stroma of infiltrating ductal adenocarcinomas of the pancreas [44]. It is formed by cancer-associated fibroblasts playing an important role in the PDAC progression [45]. Overexpression of miR-196 could be a negative feedback against further paladin production. Our findings show that the more advanced stage of PDAC, the higher (although non-significant) the expression of miR-196. Upregulation of miR-196 was associated with cell proliferation, migration and invasion, suggesting its utility in early diagnosis [46]. In addition, the latest study demonstrates that miR-196 has significant prognostic value in the future cancer development [47].

Deregulation of the cell growth is one of the most important features in neoplasia. According to miRWalk database one of the miR-200 targets is *KLF11* gene. Its product participates in cellular inflammation, differentiation and apoptosis. Under normal condition, protooncogene c-myc is suppressed by transforming growth factor beta (TGF-β) signalling which is mediated by KLF11 [48,49]. In PDAC with Ras mutation, the KLF11 protein is inactivated and thus the cell growth is deregulated. However, overexpression of miR-200 observed in PDAC could lead to direct inhibition of proteosynthesis of KLF11 and then to deregulated growth. Overexpression of miR-200 family is non-specific for PDAC and can be found in other cancers as well. Exacerbation of the growth, metastatic properties and worse prognosis were found in transformed mammary epithelial cells connected with overexpression of miR-200 family [50], as well as in early gastric cancer [51], oral squamous cell cancer [52], colorectal cancer [53], ovarian cancer [54] or in non-small cell lung cancer [55]. MiR-200 family members are potential prognostic biomarkers in patients with various carcinomas [56,58]. In experimental study in mice, the upregulation of miR-200 induced beta-cell apoptosis and lethal Type 2 diabetes [57]. A crucial role for the miR-200 family in beta-cell survival and the pathophysiology of diabetes was suggested.

The biosenzors evaluating cluster of miRNAs may represent new way for miRNA analysis in different clinical studies. New data demonstrate that cluster or panel of several miRNAs will improve diagnostic process rather than evaluation of single miRNA expression [15,31].

Limitation of our study is that only few patients were diagnosed in the stage T1+2 of the cancer to see whether the changes in the miRNA expressions are progress-dependent or if they are present just due to the malignancy. On the other hand, it reflects the current state of late diagnosis of the cancer. To validate miRNAs as early markers, the precursors of PDAC (IPMN and PanIN lesions) should be included. Recent data confirm the possibility to use panel of several miRNAs for early diagnosis of PDAC including its precursors as well as for follow up observation in chronic pancreatitis [25,31,35]. For the evaluation of miRNAs results we used relative expressions for quantification instead of absolute values.

## Conclusions

We conclude that miR-21, -196, -200 and -423 improve both sensitivity and specificity in combination with CA19-9 in detecting PDAC. Our results demonstrate a significant difference in the expression of six miRNAs which could distinguish PDAC from CP. Three miRNAs (miR-21, miR-192 and miR-200) were the best markers for PDAC. However, small number of patients with the lowest stage (T1) of PDAC was analyzed. The most impressive decrease of miR-196 and miR-423 found in CP patients could suggest their determination as markers in chronic pancreatitis. Both miRNAs could be used in long-term follow-up of patients with CP in which they would bring important information on new development of PDAC.

Further studies in the population with high risk of PDAC will be necessary to disclose the early changes of miRNA expressions associated with precursors of pancreatic cancer. Panel of selected miRNAs will be preferred to single miRNA molecule.

## Figures and Tables

**Table 1:** Characteristics of patients with pancreatic ductal adenocarcinoma (PDAC), chronic pancreatitis (CP), type 2 diabetes mellitus (T2DM) and control persons.

	PDAC (n=77)		CP (n=26)		T2DM	Controls
	PDAC DM^+^	PDAC DM^−^	CP DM^+^	CP DM^−^		
	(n=60)	(n=17)	(n=18)	(n=8)	(n=39)	(n=25)
*Age (years)*	68±8	68±10	60±11	66±10	63±6	63±7
*Sex (M/F)*	35/25	9/8	11/7	4/4	30/9	22/7
*BMI (kg/m* *^2^* *)*	25.8±5.5	26.1±4.3	24.5±3.4	24.0±2.2	30.1±4.3*	27.2±3.6
*FPG (mmol/l)*	8.7±4.1^a^	5.1±0.9	10.6±4.8^a^	5.3±0.4	7.7±1.6^a^	5.3±0.6
*HbA1c (mmol/mol)*	52.5±18.7^a^	35.7±6.5	67.4±34.4^a^	37.6±4.0	51.8±12.9^a^	37.8±4.6
*CA19-9 (kU/l)*	132.0^bc^ (68.6–253.9)	94.8^c^ (34.1–263.5)	13.1 (6.4–27.1)	9.1 (1.8–46.0)	8.1 (6.1–10.7)	4.7 (2.8–7.7)

PDAC DM^+^: patients with pancreatic adenocarcinoma with diabetes or prediabetes, PDAC DM^−^: patients with pancreatic adenocarcinoma without impaired glucose metabolism, CP DM^+^: patients with chronic pancreatitis and diabetes or prediabetes, CP DM^−^: patients with chronic pancreatitis without impaired glucose metabolism, BMI: body mass index, FPG: fasting plasma glucose. Results are expressed as means with standard deviation (SD) or geometric mean with 95 % confidence interval for CA19-9. Statistical significance as compared to CP DM^−^, CP DM^+^ and PDAC DM^+^ (* p<0.01). Significant differences in diabetic vs non-diabetic patients (^a^ p<0.005), in PDAC vs. CP patients (^b^ p<0.02) and vs. T2DM and control groups (^c^ p<0.001).

**Table 2:** Relative microRNA expressions (fold-change) according to presence/absence of diabetes mellitus in the groups compared to healthy control (expressions equal to 1).

	PDAC (n=77)		CP (n=26)		T2DM
	PDAC DM^+^	PDAC DM^−^	CP DM^+^	CP DM^−^	
	(n=60)	(n=17)	(n=18)	(n=8)	(n=39)
*miR-21*	2.2^ad^ (1.8–2.9)	2.2^ad^ (1.7–3.0)	0.9 (0.4–2.0)	0.3 (0.1–1.4)	0.6 (0.4–1.0)
*miR-30*	1.8^bd^ (1.5–2.2)	1.7^bd^ (1.1–2.4)	0.7 (0.5–1.2)	0.5 (0.2–1.2)	0.8 (0.6–1.1)
*miR-192*	3.3^ad^ (2.4–4.6)	2.5^b^ (1.9–3.3)	1.7 (1.2–2.4)	1.0 (0.6–1.9)	1.0 (0.6–1.5)
*miR-196*	1.6^bc^ (1.3–2.0)	1.9^bc^ (1.3–2.8)	0.3^a^ (0.2–0.4)	0.4^b^ (0.2–0.6)	0.8 (0.7–1.0)
*miR-200*	4.1^ad^ (2.7–6.3)	2.7^bd^ (1.4–5)	1.1 (0.9–1.5)	0.9 (0.3–2.7)	1.0 (0.8–1.3)
*miR-423*	1.3^c^ (1.1–1.5)	1.3^c^ (0.9–2.1)	0.2^a^ (0.2–0.3)	0.2^a^ (0.1–0.4)	0.9 (0.7–1.1)

PDAC DM^+^: patients with pancreatic adenocarcinoma with diabetes or prediabetes, PDAC DM^−^: patients with pancreatic adenocarcinoma without impaired glucose metabolism, CP DM^+^: patients with chronic pancreatitis and diabetes or prediabetes, CP DM^−^: patients with chronic pancreatitis without impaired glucose metabolism. Statistical significance: compared to healthy control/T2DM: ^a^ p<0.001, ^b^ p<0.01, compared to CP: ^c^ p<0.001, ^d^ p<0.01. Data are geometric mean with 95 % confidence interval.

## References

[1] Siegel RL, Miller KD, Jemal A (2018). Cancer statistics, 2018. CA Cancer J Clin.

[2] Lowenfels AB, Maisonneuve P, Cavallini G, Ammann RW, Lankisch PG, Andersen JR, Dimagno JP, International Pancreatitis Study Group (1993). Pancreatitis and the risk of pancreatic cancer. N Engl J Med.

[3] Juthani R, Manne A (2025). Blood-based biomarkers in pancreatic ductal adenocarcinoma: developments over the last decade and what holds for the future- a review. Front Oncology.

[4] Wolrab D, Jirásko R, Cífková E, Höring M, Mei D, Chocholoušková M, Peterka O (2022). Lipidomic profiling of human serum enables detection of pancreatic cancer. Nat Commun.

[5] Hasani F, Masrour M, Khamaki S, Jazi K, Hosseini S, Heidarpour H, Namazee M (2025). Diagnostic and Prognostic Accuracy of MiRNAs in Pancreatic Cancer: A Systematic Review and Meta-Analysis. J Cell Molec Med.

[6] Gayral M, Jo S, Hanoun N, Vignolle-Vidoni A, Lulka H, Delpu Y, Meulle A (2014). MicroRNAs as emerging biomarkers and therapeutic targets for pancreatic cancer. World J Gastroenterol.

[7] Al-Temaimi R, Abdulkarim B, Al-Ali A, John B, Mallik MK, Kapila K (2024). Analysis of Candidate miRNAs’ Expression in Pancreatic Cancer. Cancer Med.

[8] Przybyszewski O, Mik M, Nowicki M, Kusinski M, Mikołajczyk-Solinska M, Sliwinska A (2024). Using microRNAs Networks to Understand Pancreatic Cancer-A Literature Review. Biomedicines.

[9] Khan IA, Saraya A (2023). Circulating MicroRNAs as Noninvasive Diagnostic and Prognostic Biomarkers in Pancreatic Cancer: A Review. J Gastroint Cancer.

[10] Powrózek T, Otieno MO, Maffeo D, Frullanti E, Martinez-Useros J (2025). Blood circulating miRNAs as pancreatic cancer biomarkers: An evidence from pooled analysis and bioinformatic study. Int J Biol Macromol.

[11] Cote GA, Gore AJ, McElyea S, Heathers LE, Xu H, Sherman S, Korc M (2014). A pilot study to develop a diagnostic test for pancreatic ductal adenocarcinoma based on differential expression of select miRNA in plasma and bile. Am J Gastroenterol.

[12] Mok ETY, Chitty JL, Cox TR (2024). miRNAs in pancreatic cancer progression and metastasis. Clin Exp Metastasis.

[13] Xia T, Chen XY, Zhang YN (2021). MicroRNAs as biomarkers and perspectives in the therapy of pancreatic cancer. Molec Cell Biochem.

[14] Škrha P, Hořínek A, Pazourková E, Hajer J, Frič P, Škrha J, Andel M (2016). Serum microRNA-196 and microRNA-200 in pancreatic ductal adenocarcinoma of patients with diabetes mellitus. Pancreatology.

[15] Singh N, Khan IA, Rashid S, Rashid S, Roy S, Kaushik K, Kumar A (2024). MicroRNA Signatures for Pancreatic Cancer and Chronic Pancreatitis. Pancreas.

[16] Guo S, Qin H, Liu K, Wang H, Bai S, Liu S, Shao Z (2021). Blood small extracellular vesicles derived miRNAs to differentiate pancreatic ductal adenocarcinoma from chronic pancreatitis. Clin Transl Med.

[17] American Diabetes Association (2010). Diagnosis and Classification of Diabetes Mellitus. Diabetes Care.

[18] Bitterlich N, Schneider J (2007). Cut-off-independent Tumour Marker Evaluation Using ROC Approximation. Anticancer Res.

[19] Frič P, Šedo A, Škrha J, Busek P, Laclav M, Skrha P, Zavoral M (2017). Early detection of sporadic pancreatic cancer: time for change. Eur J Gastroenterol Hepatol.

[20] Škrha J, Frič P, Bušek P, Škrha P, Šedo A, RODRIGO (2018). Sporadic pancreatic cancer - glucose homeostasis and pancreatogenic Type 3 diabetes. Advances in Pancreatic Cancer.

[21] Lopez NE, Prendergast C, Lowy AM (2014). Borderline resectable pancreatic cancer: Definitions and management. World J Gastroenterol.

[22] Wagner M, Redaelli C, Seiler CA, Seiler CA, Friess H, Buchler MW (2004). Curative resection is the single most important factor determining outcome in patients with pancreatic adenocarcinoma. Br J Surg.

[23] Bedi MMS, Gandhi MD, Jacob G, Lekha V, Venugopal A, Ramesh H (2009). CA 19-9 to differentiate benign and malignant masses in chronic pancreatitis: is there any benefit?. Indian J Gastroenterol.

[24] Poruk KE, Gay DZ, Brown K, Mulvihill JD, Boucher KM, Scaife CL, Firpo MA, Mulvihill SJ (2013). The Clinical Utility of CA 19-9 in Pancreatic Adenocarcinoma: Diagnostic and Prognostic Updates. Curr Mol Med.

[25] Kawai M, Fukuda A, Otomo R, 38 co-authors (2024). Early detection of pancreatic cancer by comprehensive serum miRNA sequencing with automated machine learning. Br J Cancer.

[26] Franklin O, Jonsson P, Billing O, Lundberg E, Öhlund D, Nyström H, Lundin C, Antti H, Sund M (2018). Plasma Micro-RNA Alterations Appear Late in Pancreatic Cancer. Ann Surg.

[27] Xue Y, Abou Tayoun AN, Abo KM, Pipas JM, Gordon SR, Gardner TB, Barth RJ (2013). MicroRNAs as diagnostic markers for pancreatic ductal adenocarcinoma and its precursor, pancreatic intraepithelial neoplasm. Cancer Genet.

[28] Slater EP, Strauch K, Rospleszcz S, Ramaswamy A, Esposito I, Klöppel G, Matthäi E (2014). MicroRNA-196a and -196b as Potential Biomarkers for the Early Detection of Familial Pancreatic Cancer. Transl Oncol.

[29] Hernandez YG, Lucas AL (2016). MicroRNA in pancreatic ductal adenocarcinoma and its precursor lesions. World J Gastrointest Oncol.

[30] Ke RS, Lv LZ, Zhang SY, Zhang FX, Jiang Y (2020). Functional mechanism and clinical implications of MicroRNA-423 in human cancers. Cancer Med.

[31] Zhou Y, Liu Y, Zong Z, Huang H, Liang L, Yang X, Xin M (2025). Rapid and sensitive detection of exosomal microRNAs by terahertz metamaterials. Spectrochim Acta A Mol Biomol Spectrosc.

[32] Cao Z, Liu C, Xu JW, You L, Wang C, Lou W, Sun B (2016). Plasma microRNA panels to diagnose pancreatic cancer: Results from a multicenter study. Oncotarget.

[33] Le Large TYS, Meijer LL, Prado MM, Kazemier G, Frampton AE, Giovannetti E (2015). Circulating microRNAs as diagnostic biomarkers for pancreatic cancer. Expert Rev Mol Diagn.

[34] Liu J, Gao J, Yiqi D, Li Z, Ren Y, Gu J, Wang X (2012). Combination of plasma microRNAs with serum CA19-9 for early detection of pancreatic cancer. Int J Cancer.

[35] Khan IA, Rashid S, Singh N, Rashid S, Singh V, Gunjan D, Das P (2021). Panel of serum miRNAs as potential 3on invasive biomarkers for pancreatic ductal adenocarcinoma. Sci Rep.

[36] Flammang I, Reese M, Ströse AJ, Yang Z, Eble JA, Dhayat SA (2020). Tumor-Suppressive miR-192-5p Has Prognostic Value in Pancreatic Ductal Adenocarcinoma. Cancers.

[37] Azadinejad H, Farhadi Rad M, Babaeizad A, Samadi A (2025). MicroRNA profiling in pancreatic cancer and chronic pancreatitis: Novel insights and pathway analysis. Human Gene.

[38] Luan X, Wang X, Bian G, Li X, Gao Z, Liu Z, Zhang Z (2025). Exosome applications for the diagnosis and treatment of pancreatic ductal adenocarcinoma: An update (Review). Oncol Rep.

[39] Xu B, Chen Y, Peng M, Zheng JH, Zuo Ch (2023). Exploring the potential of exosomes in diagnosis and drug delivery for pancreatic ductal adenocarcinoma. Int J Cancer.

[40] Tiwari PK, Shanmugam P, Karn V, Gupta S, Mishra R, Rustagi S, Chouhan M (2024). Extracellular Vesicular miRNA in Pancreatic Cancer: From Lab to Therapy. Cancers.

[41] Wlodarczyk B, Durko L, Walczak K, Talar-Wojnarowska R, Malecka-Wojciesko E (2024). Select Endocrine Disorders and Exosomes in Early PDACDiagnosis. Int J Mol Sci.

[42] Otey CA, Rachlin A, Moza M, Arneman D, Carpen O (2005). The Palladin/Myotilin/Myopalladin Family of Actin-Associated Scaffolds. Int Rev Cytol.

[43] Pogue-Geile KL, Chen R, Bronner MP, Crnogorac-Jurcevic T, Moyes KW, Dowen S, Otey CA (2006). Palladin Mutation Causes Familial Pancreatic Cancer and Suggests a New Cancer Mechanism. PLoS Med.

[44] Salaria SN, Illei P, Sharma R, Walter KM, Klein AP, Eshleman JR, Maitra A (2007). Palladin is overexpressed in the non-neoplastic stroma of infiltrating ductal adenocarcinomas of the pancreas, but is only rarely overexpressed in neoplastic cells. Cancer Biol Ther.

[45] Sato D, Tsuchikawa T, Mitsuhashi T, Hatanaka Y, Marukawa K, Morooka A, Nakamura T (2016). Stromal Palladin Expression Is an Independent Prognostic Factor in Pancreatic Ductal Adenocarcinoma. PLoS One.

[46] Alvarez-Teijeiro S, Menendez ST, Angeles Villaronga M, Rodrigo JP, Manterola L, de Villalaín L, de Vicente JC (2017). Dysregulation of Mir-196b in Head and Neck Cancers Leads to Pleiotropic Effects in the Tumor Cells and Surrounding Stromal Fibroblasts. Sci Rep.

[47] Xiong M, Wang P, Pan B, Nie J, Wang S, He B (2021). The diagnostic and prognostic values of microRNA-196a in cancer. Biosci Rep.

[48] Buck A, Buchholz M, Wagner M, Adler G, Gress T, Ellenrieder V (2006). The tumor suppressor KLF11 mediates a novel mechanism in transforming growth factor beta-induced growth inhibition that is inactivated in pancreatic cancer. Mol Cancer Res.

[49] Ellenrieder V, Buck A, Harth A, Jungert K, Buchholz M, Adler G, Urrutia R, Gress TM (2004). KLF11 mediates a critical mechanism in TGF-beta signaling that is inactivated by Erk-MAPK in pancreatic cancer cells. Gastroenterology.

[50] Sanchez-Cid L, Pons M, Lozano JJ, Rubio N, Guerra-Rebollo M, Soriano A, Paris-Coderch L (2017). MicroRNA-200, associated with metastatic breast cancer, promotes traits of mammary luminal progenitor cells. Oncotarget.

[51] Yu L, Wu D, Gao H, Balic JJ, Tsykin A, Han TS, Liu YD (2018). Clinical utility of a STAT3-regulated microRNA-200 family signature with prognostic potential in early gastric cancer. Clin Cancer Res.

[52] Arunkumar G, Rao AKDM, Manikandan M, Rao HPS, Subbiah S, Ilangovan R, Murugan AK, Munirajan AK (2018). Dysregulation of miR-200 family microRNAs and epithelial-mesenchymal transition markers in oral squamous cell carcinoma. Oncol Lett.

[53] O’Brien SJ, Carter JV, Burton JF, Oxford BG, Schmidt MN, Hallion JC, Galandiuk S (2018). The role of the miR-200 Family in Epithelial-Mesenchymal Transition in Colorectal Cancer: A Systematic Review. Int J Cancer.

[54] Koutsaki M, Libra M, Spandidos DA, Zaravinos A (2017). The miR-200 family in ovarian cancer. Oncotarget.

[55] Si LB, Tian H, Yue WM, Li L, Li SH, Gao C, Qi L (2017). Potential use of microRNA-200c as a prognostic marker in non-small cell lung cancer. Oncol Lett.

[56] Lee JS, Ahn YH, Won HS, Sun DS, Kim YH, Ko YH (2017). Prognostic Role of the MicroRNA-200 Family in Various Carcinomas: A Systematic Review and Meta-Analysis. Biomed Res Int.

[57] Belgardt BF, Ahmed K, Spranger M, Latreille M, Denzler R, Kondratiuk N, von Meyenn F (2015). The microRNA-200 family regulates pancreatic beta cell survival in type 2 diabetes. Nat Med.

[58] Lohajová Behulová R, Bugalová A, Bugala J, Struhárňanská E, Šafranek M, Juráš I (2023). Circulating Exosomal miRNAs as a Promising Diagnostic Biomarker in Cancer. Physiol Res.

